# Supplementary figures and images for: The role of health education on cervical cancer screening uptake at selected health centers in Addis Ababa
Source: PLoS One. 2020 Oct 7;15(10):e0239580. doi: 10.1371/journal.pone.0239580 (PMC7540882; doi:10.1371/journal.pone.0239580)

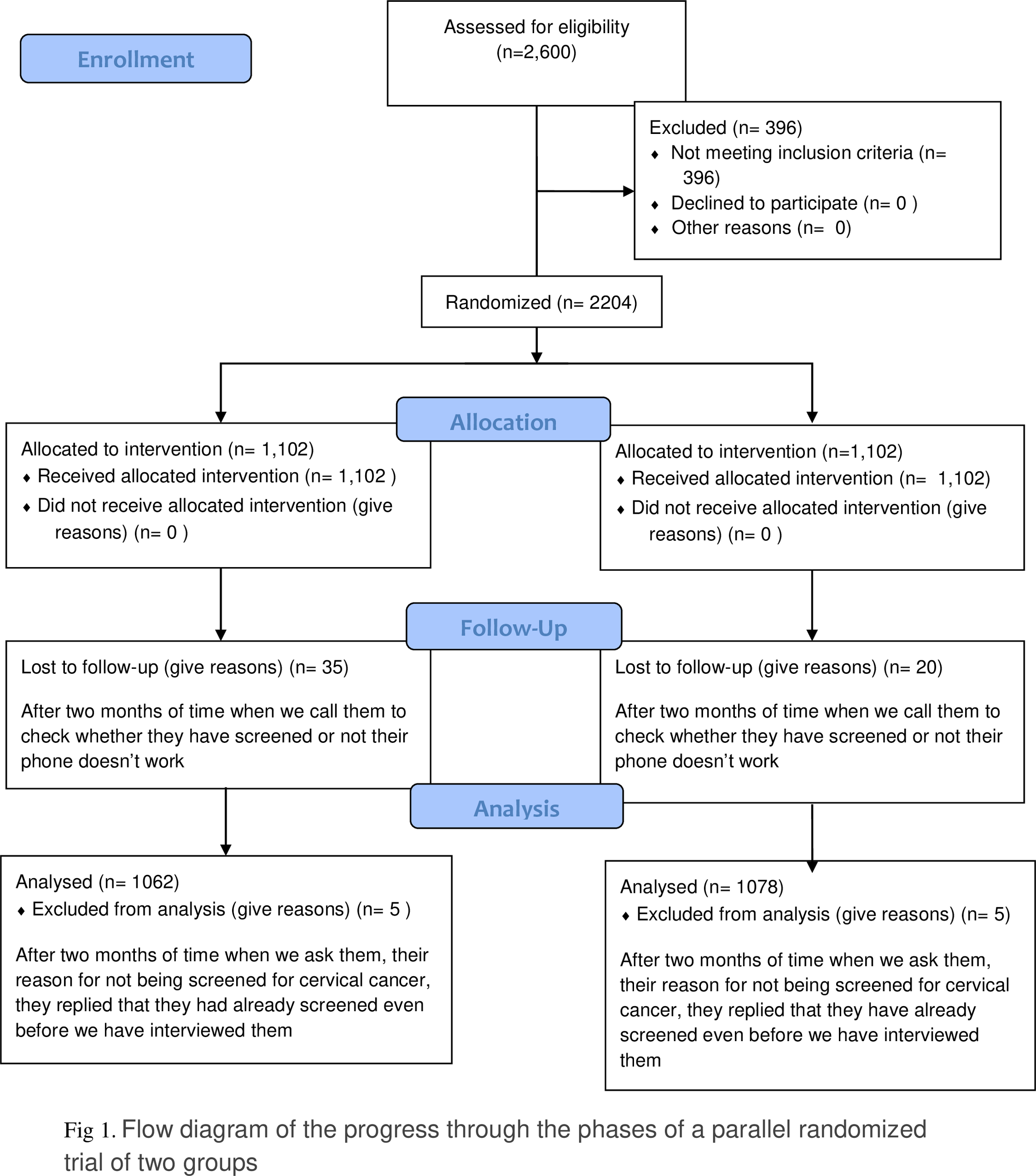

Supplement: S1 Fig — (TIF) [file pone.0239580.s001.tif]
