# Supplementary material for: The role of health education on cervical cancer screening uptake at selected health centers in Addis Ababa
Source: PLoS One. 2020 Oct 7;15(10):e0239580. doi: 10.1371/journal.pone.0239580 (PMC7540882; doi:10.1371/journal.pone.0239580)
Supplement: S1 File — (DOC) [file pone.0239580.s002.doc]

| **Assessment of the impact of Health Information Dissemination and Print Media to Enhance Cervical Cancer Screening In Addis Ababa; Cluster Randomized Controlled trial** |
| --- |
| **Research proposal**    January, 2017 |

Table of Contents

[Summary v](#__RefHeading___Toc24962562)

[Introduction 6](#__RefHeading___Toc24962563)

[Back ground 6](#__RefHeading___Toc24962564)

[Statement of the problem 7](#__RefHeading___Toc24962565)

[Significance of the proposed study 8](#__RefHeading___Toc24962566)

[Literature review 8](#__RefHeading___Toc24962567)

[Cervical cancer 8](#__RefHeading___Toc24962568)

[Methods for cervical cancer screening 9](#__RefHeading___Toc24962569)

[Practice and Barriers of Cervical Cancer Screening 10](#__RefHeading___Toc24962570)

[Contribution of health information dissemination for cervical cancer screening 10](#__RefHeading___Toc24962571)

[Objectives 11](#__RefHeading___Toc24962572)

[General objective 11](#__RefHeading___Toc24962573)

[Specific Objectives 11](#__RefHeading___Toc24962574)

[Methods 12](#__RefHeading___Toc24962575)

[Study area 12](#__RefHeading___Toc24962576)

[Study period 13](#__RefHeading___Toc24962577)

[Study design- 13](#__RefHeading___Toc24962578)

[Source population- 13](#__RefHeading___Toc24962579)

[Study population 13](#__RefHeading___Toc24962580)

[Sampling Selection Procedures 15](#__RefHeading___Toc24962581)

[Operational definition 16](#__RefHeading___Toc24962582)

[Data processing and analysis: 16](#__RefHeading___Toc24962583)

[Pre- test 16](#__RefHeading___Toc24962584)

[Data quality control: 16](#__RefHeading___Toc24962585)

[Data collection tools 17](#__RefHeading___Toc24962586)

[Data collectors: 17](#__RefHeading___Toc24962587)

[Ethical consideration: 17](#__RefHeading___Toc24962588)

[Dissemination: 17](#__RefHeading___Toc24962589)

[Work plan 18](#__RefHeading___Toc24962590)

[BUDGET 19](#__RefHeading___Toc24962591)

[References 21](#__RefHeading___Toc24962592)

[Annexes 24](#__RefHeading___Toc24962593)

# List of abbreviation/ Acronyms

# AAHB Addis Ababa Health Bureau

# AAU Addis Ababa University

# CC Cervical Cancer

# CSA Central Statistical Agency

# FMOH Federal Ministry of Health

# HPV Human Papilloma Virus

# VIA Visual Inspection with Acetic Acid

# WHO World Health Organization

# Summary

**Introduction**- Cervical cancer is the most preventable cancer from all reproductive organ cancers. There were about 7,095 newly diagnosed cervical cancer cases in 2012 in Ethiopia. A study done at Black Lion Hospital among cervical cancer patients revealed that only 3% of study participants were ever screened for cervical cancer before diagnosis. Considering oneself as healthy and no need for screening were mentioned as reasons for not being screened. These evidences clearly indicate that, lack of knowledge is associated with low uptake of cervical cancer screening.

**Objective**-This study aims to determine the joint effect of health information dissemination and print media on seeking cervical cancer screening service among women who visit health centers in Addis Ababa.

**Methodology**- This clustered randomized controlled trial, will be conducted in eight selected health centers in Addis Ababa. Visual Inspection with acetic acid, one of cervical cancer screening methods is available in all eight health centers. Four health centers will be randomized to the intervention group and another four to a control group. The estimated total sample size shall be 2,203. The study participants will be mothers in the age range of 30-49 years who have no previous history of cervical cancer or never been screened before. Participants will be recruited when they visit the selected health centers. The intervention is health education and print media concerning cervical cancer screening. Practice of cervical cancer screening will be assessed; after 2 months of time from recruitment period. In the four control health centers, no intervention will take place. Data both from the intervention and control facilities will be collected before and after the intervention. Appropriate statistical analysis will be conducted using STATA version 14 statistical software. Ethical clearance will be obtained from Addis Ababa University College of Health Sciences Institutional Review Board and Addis Ababa health bureau.

**Expected outcome-** This study will contribute for designing the best way of intervention at primary health centers to increase the uptake of cervical cancer screening

| IntroductionBack ground Cervical cancer is one of the most common and preventable types of cancers among women (1). Human papilloma virus (HPV) infection is the most common risk factor for cervical cancer (1). The other risk factors include smoking, past or current chlamydial infection , having diet low in fruits and vegetables, being overweight and long term use of oral contraceptives (2). Women with early cervical cancer and precancerous lesion do not usually present with signs and symptoms. But when the cancerous lesion involve other tissues and becomes invasive cancer, different symptoms like vaginal bleeding, pain during intercourse and vaginal discharge can be observed (2).  Screening prevents cervical cancer through the identification of pathologic changes in cervical tissue. Through screening, pre-cancerous lesion can be detected and if treated progress of invasive cervical cancer can be halted. Visual inspection with acetic acid (VIA) screening method is especially recommended by WHO for a country like Ethiopia where there are limited facilities. Visual inspection with acetic acid (VIA) screening method is simple, inexpensive, needs low technology and minimal infrastructure for use (3). |
| --- |

## Statement of the problem

In 2012, there were an estimated 528,000 new cases of cervical cancer worldwide (4).

According to Globocan, there were about 7,095 newly diagnosed cancer cases in 2012 in Ethiopia, accounting for 17.3% of total cancer cases in females (4). Cervical cancer death is common in populations where there is no routine cervical cancer screening service and is a major cause of cancer death among women in developing countries (5). Due to lack of information, more than 80% of cervical cancer cases in sub Saharan Africa are detected at late stage (6). Late stage of the disease is associated with low survival rate**.** In Ethiopia annually an estimated 3,235 deaths due to cervical cancer occurs (6).

Cervical cancer screening can effectively reduce mortality from the disease (3). However population-based surveys indicated that coverage of cervical cancer screening in developing countries is low (19%), compared to 63% in developed countries (7). A study done in Nigeria reveled that only 1.4 % from 2,000 women ever practiced cervical cancer screening and the main reason mentioned for not screening by 95.5% of the study participants was lack of awareness (8). In another study in Kenya, only 16.6 % of the study participants were screened for cervical cancer and from those ever screened, only 4% screened more than once in their life time (9). A study done at Black Lion Hospital among cervical cancer patients, only 3% of study participants ever screened for cervical cancer before diagnosis (10). Considering oneself as healthy and no need for screening, lack of awareness about screening services and fear of screening tests were mentioned reasons for not screening (9). These evidences clearly indicate that, lack of knowledge is associated with low uptake of cervical cancer screening.

Even though multiple studies have indicated low level of knowledge for cervical cancer screening, a challenge still exists in identifying the best way to create awareness of cervical cancer screening for the women’s in reproductive age.

One of the strategies recommended by FMOH to increase use of cervical cancer screening service is one to one education for women. However, one to one education may be difficult to implement in a setting where there is high patient flow and limited health service providers. So, this study assesses the contribution of health information dissemination and print media to enhance cervical cancer screening service utilization in Addis Ababa.

## Significance of the proposed study

The outcome of this study will contribute for designing the best way of intervention at primary health centers to increase the uptake of cervical cancer screening. Moreover, the finding of this study can serve as a baseline for other researchers to investigate more in the area and organizations that work in the area will also benefit from the finding

# Literature review

## Cervical cancer

Cervical cancer is one of the most common types of cancer among women and it is the most preventable type of cancer. Squamous cell carcinoma and adenocarcinoma are the two main types of cervical cancer and squamous cell carcinoma is commonest type from the two (11). It is a complication of Human Papilloma Virus (HPV) infection (3). This virus is mainly transmitted sexually; it can also be transmitted through direct skin to skin contact of the genital areas (12). The most common symptom of cervical cancer is abnormal vaginal bleeding. Even though cervical cancer is a deadly disease once it reaches the invasive stage, out of all female genital tract cancers it is the only preventable cancer in the early stage (13).

From women cancer worldwide, cervical cancer accounts the 4^th^ most common cancer with an estimated 528,000 new cases in 2012 (4). There were estimated 266,000 deaths from cervical cancer; 87% of deaths related to cervical cancer occur in less developed regions (2). In Germany, there are 4,495 new cervical cases diagnosed every year (14). In Tanzania, cervical cancer is the leading cause of cancer among women age 15-44 years and it is estimated that 7,302 cases occur annually (15). Every year in Ghana, 3052 women are diagnosed with cervical cancer and 1,556 women die from the disease (16). The majority of cervical cancer mortality and morbidity occur in women who have never been screened or treated and in women with well-described sexual and reproductive risk factors, such as an early sexual debut, a history of multiple sexual partners, and a high number of live births and use of hormonal contraceptives.

## Methods for cervical cancer screening

The best method to diagnose cervical cancer at early stage or before it becomes invasive cervical cancer is through regular cervical cancer screening. Screening programs are effective if the cancer is one of the most common types for which there are cost effective, affordable, accessible and acceptable screening methods (17). Several screening methods are used to detect precancerous lesion and cancer of the cervix. These screening methods are available, and can be performed safely and inexpensively in an outpatient setting (7). VIA screening method uses house hold vinegar and if there is precancerous lesion, it turns to white within few minutes. Which creates an opportunity for trained health professionals to treat it immediately and this screening method is single visit approach which is beneficial in many ways for the patient (18).

For cervical cancer possible screening methods are visual inspection with acetic acid (VIA), HPV testing for cervical cancer; PAP cytology test for cervical cancer in middle- and high-income settings (17).

Because of simple cervical cancer screening known as “Pap test”, deaths related with cervical cancer decreased by 75 % in developed countries (18). Once, for American women, cervical cancer was one of the most common causes of death, but within the last 40 years death rate for cervical cancer decreased by over 50 % (2). This success is achieved by one of methods for cervical cancer screenings, which is Pap test (2).

In Ethiopia, prior to the introduction of pre- cancer treatment of cervical cancer, screening was not effective since treatment could not be done immediately (10). Currently, because of the availability of Cryotherapy, treatment can be done immediately after cervical cancer screening, if the test is positive.

## Practice and Barriers of Cervical Cancer Screening

A survey done in the United States among 133, 8521 women age b/n 21-65 years reveled that 11.4% had not been screened for cervical cancer in the past five years (19). Due to educational level of the society, availability of screening methods and other related factors, cervical cancer screening practice has difference in developed and undeveloped countries

Based on the study in Nepal, only 18.1 % and 10.5 % had knowledge and uptake of Pap test; respectively (20). A study done among 514 Zimbabwean Women revealed that only 9% ever practiced cervical cancer screening and identified barriers for not practicing cervical cancer screening were, lack of knowledge and advice from health professional about cervical cancer screening (21) .

The practice of cervical cancer screening practice is low and in middle and low income countries (22). The study done among 1186 age eligible women in Mekelle revealed that only 19.8% have history of cervical cancer screening and absence of symptoms is major reason mentioned for not using cervical cancer screening service (22). A qualitative study done in Arbaminch town showed that, lack of awareness, not considering oneself as susceptible for cervical cancer, fear of the test and result, potential shame if diagnosed with cervical cancer were mentioned barriers for cervical cancer screening (23). Females who had prior knowledge of cervical screening tests were 83% more likely to access cervical screening compared to those who had no prior knowledge (21).

## Contribution of health information dissemination for cervical cancer screening

Community based educational programs increase knowledge about cervical cancer and improves health seeking behavior for cervical cancer screening (24). Health education concerning cervical cancer screening is basic for early detection of the disease and it is important that educational programs should be in the context of culture of the country (3). A randomized controlled trial revealed that, 73.2% of women who received health education had Pap smear test after the intervention and were more likely to answer almost all knowledge item questions correctly (25). A study in Nigeria showed that educational interventional program has effect on increasing awareness of cervical cancer and uptake of Pap smear test (21). From 2010-2014 ; 16,632 HIV positive women who were counseled about cervical cancer screening using single visit approach and 99% of them were screened using VIA (26).

The FMoH in Ethiopia recommends education for women who never have cervical cancer screening. The three basic strategies recommended by the FMoH to educate women who never have cervical cancer screening are (6) –

1. Facility based teaching which can be one to one or group education which addresses individuals that come to health facilities.
2. Media based – Using radio/television or print media to large dispersed communities
3. mHealth and eHealth- Health education using mobile and Internet technologies (6).

# Objectives

## General objective

To determine the joint effect of health information dissemination and print media on seeking cervical cancer screening service among women who visit health centers in Addis Ababa.

## Specific Objectives

- To determine the effect of health education on cervical cancer screening uptake at the health centers of Addis Ababa

**Secondary objective**

- To describe the outcomes of VIA screening at the health centers of Addis Ababa

# Methods

## Study area

The study will be conducted in Addis Ababa, ten selected health centers. Addis Ababa is the capital city of Ethiopia. According to the 2013 Central statistical Authority population projection report for July 2016, there are about 3.35 million people residing in Addis Ababa. The number of females in reproductive age group constitutes 34.53% of total population (27). The city has ten sub-cities and 116 districts. There are 5 hospitals owned by Addis Ababa health bureau, 4 by Federal Ministry of Health and 1 by Addis Ababa University, 3 by non-governmental Organization, 3 by defense force and police and 34 by private owners. There are 96 public health centers and around 700 private clinics out of which 75 are higher clinics. The study will be conducted at ten selected health centers in Addis Ababa.

Study period **-** The study will be conducted from June 2017- Nov. 2017

Study design- Two arm cluster randomized controlled trial

**Study population**

Source population- Women in the age range of 30-49 years in Addis Ababa who visit government health centers for immunization service for their babies.

Study population – Mothers in the age range of 30-49 years who fulfill the eligibility criteria and will come to the selected health facilities for 10^th^ week immunization schedule for their babies during the study period.

| **Variable** | **Power** | **Level of significance** | **P1** | **P2** | **N- total** |
| --- | --- | --- | --- | --- | --- |
| Proportion of women screened for cervical cancer | 90% | 5% | 10% | 15% | 1,836 |

**Sample Size calculation**

There will be 8 clusters with the size of 230

P1- Proportion of women screened for cervical cancer at the control health centers

P2- Expected proportion of women screened for cervical cancer at the intervention health centers

1,836 + 20% lost to follow up - 2,203

## Sampling Selection Procedures

Health centers that provide VIA screening service(n=14). From this list 8 health centers will be selected based on high VIA screening load. Randomization will be done to assign Intervention and Control sites. Four health centers will be assigned as intervention and 4 as control health centers

- **Intervention group**- Mothers who are in the age range of 30-49 years who will visit the selected 4 health centers to have their babies immunized at their 10^th^ week will be included. Health education based on health belief model. The health education will be Addressed by one to one brief heath talk by the health provider and educational brochure. After two months of time, it will be checked whether the women is screened for cervical cancer or not
- **Control group**- Women in the age range of 30-49 years who will visit immunization service for 10^th^ weeks of vaccination of their babies at the study areas will be included. At first contact some socio demographic variables will be taken and the women will be requested whether she has screened cervical cancer or not after two months of time from the recruitment period .

***Inclusion criteria***

- Women who live in Addis Ababa

- Women in the age range of 30-49 years

- Women who have no history of cervical cancer screening and not diagnosed for

Cervical cancer.

Variables

- Proportion of women screened
- Socio demographic and health related variables

## Operational definition

**Practice of cervical cancer screening**- Those individuals who are screened for cervical cancer using VIA method after they are recruited as study participants

## Data processing and analysis:

- Data entry and analysis will be done using Epidata version 3.1 and STATA 14; respectively
- Descriptive analysis- Frequency, Proportion, mean, and standard deviation
- Chi- square test (level of significance 5%) will be employed to check the statistical significance between the proportion of women screened at the intervention and control health centers

Pre- test The interview questionnaire will be pretested on 5 % of the sample size to make it more reliable and context oriented. The pre- test questionnaire will not be used in the main study. Those who will participate in the pretest will give their feedbacks about the education given about cervical cancer screening, the instrument for data collection and the interview.

Data quality control: The interview questionnaire will be pretested to make further adjustments if need be so that the tool will be reliable enough to make valid measurements. Data collectors will have adequate training on the tools and interview techniques to enhance their interview skills, familiarize them with the measurement tools and enable them understand the meaning and implication of every variable in the questionnaire. Data collectors will submit the data every day for the respective supervisors to be checked for completeness and errors. After the data are checked for completeness, it will be cleaned during and after entry. Before data entry a template with define legal values and skipping patterns will be prepared by using Epi Data statistical software to decrease possible errors during data entry. After data entry, SPSS statistical software will be used to check for outlying values. During data collection there will be close field supervision to quickly deal with circumstances that may affect data quality.

Data collection tools**:** Structured Interview questionnaire will be used.

Data collectors: Ten data collectors who have diploma/degree in nursing who gives maternal and child care service will be recruited. The data collectors will be individuals who are working in the selected study areas. Two supervisors will check the collected data for completeness and reliability regularly.

Ethical consideration: Ethical clearance will be obtained from the Addis Ababa University College of Health Sciences Institutional Review Board and Addis Ababa Health Bureau. Letter of support will be also obtained from the Addis Ababa Health Bureau. Permission will be obtained from each study area. Each respondent will be informed about the purpose, scope and expected outcome of the research, written informed consent will be obtained from study participants prior to their enrolment. Anyone who is not willing to participate will be excluded from the study; and during the interview, respondents who are interested to avoid specific questions or discontinue the interview will be allowed to do so.

Dissemination: Final result of the study will be presented to the Addis Ababa University, Federal Ministry of Health, Addis Ababa Health Bureau (AAHB) and other stake holders. Eventually, the findings will be published in peer reviewed international and reputable national journals.

# Work plan

**Table2. Work plan 2017.**

| **No** | **Tasks to be performed** | **Responsible person** | **2017** | | | | | | | | |
| --- | --- | --- | --- | --- | --- | --- | --- | --- | --- | --- | --- |
|  |  |  | **Apr.** | **May.** | | | **June** | **July** | **August** | **Sept.** | **Oct.** |
| 1- | Ethical clearance | A.A.U. IRB |  |  | | |  |  |  |  |  |
| 2- | Recruiting data collectors | Research team |  |  |  | |  |  |  |  |  |
| 3- | Training of data collectors | Research team |  |  |  |  |  |  |  |  |  |
| 4- | Pre- test | Research team |  |  |  |  |  |  |  |  |  |
| 5- | Data collection and Health education for Intervention group | Data collectors |  |  | | |  |  |  |  |  |
| 7- | Second phase data collection | Data collectors |  |  | | |  |  |  |  |  |
| 8- | Data analysis and write up | Investigators |  |  | | |  |  |  |  |  |
| 12 | Dissemination | Investigators |  |  | | |  |  |  |  |  |

# BUDGET

| **Item** | **Unit price** | **Total price**  **(Birr)** | **Remark** |
| --- | --- | --- | --- |
| **Personnel** | | | |
| Data collectors payment | 656*2*50 | 65,600 birr | Data will be collected twice and the sample size is 656. Per questionnaire 50 birr will be paid for data collectors. |
| Per diem for data collectors | 500*2*5 | 5,000 birr | For 5 data collectors in the intervention group who will teach women about cervical cancer screening |
| Per diem for data collectors during training | 300*10*2 | 6000 birr | The training will be for two days |
| Per diem for trainers of data collectors | 500*2*3 | 3,000 birr | There will be two trainers |
| Secretary per- diem | 200*10days | 2000 birr |  |
| Supervisors perdiem | 3000*3*4 | 36,000 birr | There will be three supervisors for 10 health centers. |
| Driver per-diem | 100*30 days | 3000 birr |  |
| Accountant top up |  | 2,000 birr |  |
| Data entry clerk | 1352*5 | 6760 birr | Per questionnaire 5 birr will be paid ( Total number of questionnaire is 1, 352) |
| **Total – 129,360 birr** | | | |
| **Logistics** | | | |
| Fuel |  | 2,000 |  |
| Communication cost ( Mobile card ) |  | 3,000 birr |  |
| Educational Materials | 300*10 | 3000 birr |  |
| Questionnaire duplication | 1352*10 | 13,520 birr |  |
| **Total – 21,520** | | | |
| **Gran total –** **150,880** **Birr** | | | |

# References

1. WHO/ICO Information Centre, 2015. HPV and Cervical Cancer and Related Cancers in Ethiopia.
2. American Cancer Society (ACS), 2016. Cervical Cancer. Www...cancer.org
3. WHO, 2002. Cervical Cancer Screening in Developing Countries: Report of a WHO Consultation. Geneva: World Health Organization.
4. GLOBOCAN 2012: Estimated cancer incidence, mortality and prevalence worldwide in 2012. <http://globocan.iarc.fr/Pages/burden_sel.aspx>
5. American Cancer Society, 2016. The American Cancer Society guidelines for the prevention and early detection of cervical cancer.
6. Federal Democratic Republic of Ethiopia, 2015. Ministry of Health, Guideline for cervical cancer prevention and control in Ethiopia.
7. Chelimo C, Wouldes TA, Cameron LD, Elwood JM, 2013. Risk factors for and prevention of human papillomaviruses (HPV), genital warts and cervical cancer.
8. Abiodun O.A, Fatungase O.K, Olu-Abiodun O.O, Idowu-Ajiboye B.A and Awosile J.O,2013. An assessment of women’s awareness and knowledge about cervical cancer and screening and the barriers to cervical screening in Ogun State, Nigeria. IOSR Journal of Dental and Medical Sciences (IOSR-JDMS) e-ISSN: 2279-0853, p-ISSN: 2279-0861. Volume 10, Issue 3 . PP 52-58. [www.iosrjournals.org](http://www.iosrjournals.org/)
9. Robert M, Julius K, Taratisio N, Faith. M, 2016. Challenges of Cervical Cancer Screening Among Women of Reproductive Age in Kisii Town, Kisii County, Kenya.** **Science Journal of Public Health** **Vol. 4, No. 4, pp. 289-296. doi: 10.11648/j.sjph.20160404.14
10. Tadesse S, 2015. Preventive Mechanisms and Treatment of Cervical Cancer in Ethiopia. Cervical Cancer 1: 101. doi:10.4172/ccoa.1000101
11. American Cancer Society (ACS) ,2016. Cervical Cancer . www..cancer.org
12. WHO, Department of Reproductive Health and Research, 2007. Cervical Cancer, human papillomavirus (HPV) and HPV vaccines, key points for policymakers and health professionals. World Health Organisation, Geneva
13. Blumenthal, P, Gaffikin, L., 2013. Cervical cancer prevention: making programs more appropriate and pragmatic. J Infect.;66(3):207–217
14. Bruni L, Barrionuevo-Rosas L, Albero G, Serrano B, Mena M, Gómez D, Muñoz J, Bosch FX, de Sanjosé S. ICO Information Centre on HPV and Cancer (HPV Information Centre), 2016. Human Papillomavirus and Related Diseases in Germany. Summary Report.
15. Bruni L, Barrionuevo-Rosas L, Albero G, Serrano B, Mena M, Gómez D, Muñoz J, Bosch FX, de SanjoséS. ICO Information Centre on HPV and Cancer (HPV Information Centre),2016. Human Papillomavirus and Related Diseases in Tanzania. Summary Report
16. ICO Information Centre on HPV and Cancer, 2016. Ghana Human Papillomavirus and Related Cancers, Fact Sheet . [WWW.HPV](http://WWW.HPV/) center.net
17. WHO, 2015. Fact sheet for Cancer. <http://www.who.int/mediacentre/factsheets/fs297/en/>
18. National Cervical Cancer Coalition (NCCC), 2016. International cervical Cancer. <http://www.nccc-online.org/about-nccc/international-cervical-cancer/>
19. Vicki B, Cheryll C, Jessica K, Greta M. , 2014. Morbidity and Mortality Weekly Report

Vital Signs: Cervical Cancer Incidence, Mortality, and Screening United States, 2007–

2012. Vol.63

1. Shrestha J, Saha R, Tripathi N.,2013. Knowledge, Attitude and Practice regarding Cervical Cancer Screening Amongst Women visiting Tertiary Centre in Kathmandu, Nepal. Nepal Journal of Medical sciences;2(2):85-90.
2. Sylvia C, Carolyn M et.al, 2011. Knowledge, Attitudes, and Demographic Factors Inﬂuencing Cervical Cancer Screening Behavior of Zimbabwean Women JOURNAL OF WOMEN’S HEALTH. Volume 20, Number 6, DOI: 10.1089/jwh.2010.2062
3. [Hinsermu](http://www.ncbi.nlm.nih.gov/pubmed/?term=Bayu H%5BAuthor%5D&cauthor=true&cauthor_uid=26963098). B, [Yibrah](http://www.ncbi.nlm.nih.gov/pubmed/?term=Berhe Y%5BAuthor%5D&cauthor=true&cauthor_uid=26963098) .B.,[Amlaku](http://www.ncbi.nlm.nih.gov/pubmed/?term=Mulat A%5BAuthor%5D&cauthor=true&cauthor_uid=26963098). M, and Amare. A, 2016. Cervical Cancer Screening Service Uptake and Associated Factors among Age Eligible Women in Mekelle Zone, Northern Ethiopia, 2015: A Community Based Study Using Health Belief Model. [PLoS One](http://www.ncbi.nlm.nih.gov/pmc/journals/440/) v.11(3)
4. Gebru Z, Gerbaba M, Dirar A, 2016. Barriers to Cervical Cancer Screening in Arba Minch Town, Southern Ethiopia: A Qualitative Study. J Community Med Health 6:401. doi:10.4172/2161-0711.1000401
5. I. Agurto, S. Arrossi, S. White, P. Coffey, I. Dzuba, A. Bingham, J. Bradley, R. Lewis, 2005. Involving the community in cervical cancer prevention program. International Journal of Gynecology and Obstetrics 89, S38—S45.
6. Mark. D, Robert. M, Karen. B, Bradley. W, Douglas. C, Penny. S, Stephen. D, Joseph K, Richard. P, 1996. Effectiveness of Health Education to Increase Screening for Cervical Cancer among Eastern-Band Cherokee Indian Women in North Carolina. JNCI J Natl Cancer Inst  88(22): 1670-1676.doi: 10.1093/jnci/88.22.1670
7. Shiferaw N, Salvador-Davila G, Kassahun K, Brooks MI, Weldegebreal T, Tilahun Y, et al.., 2016. The single-visit approach as a cervical cancer prevention strategy among women with HIV in Ethiopia: successes and lessons learned. Glob Health Sci Pract.2016;4(1):87-98. <http://dx.doi.org/10.9745/GHSP-D-15-00325>.
8. Finance and Economic Development Bureau Population Affairs Coordination Sub process, 2009 ADDIS ABABA POPULATION IMAGES, 2009 Population Affairs Coordination Sub process Finance and Economic Development Bureau.

# Annexes

**አዲስ አበባ ዩኒቨርስቲ**

**ጤና ሳይንስ ኮሌጅ**

**የሕብረተሰብ ጤና ትምህርት ቤት**

እነዚህን አራት ጥያቄዎች ይጠይቁ

| **ተ.ቁ** | **መስፈርት** | **አዎ** | **አይደለም** |
| --- | --- | --- | --- |
| 1 | እድሜ ከ30-49 |  |  |
| 2 | ፅሁፍ ማንበብ ትችያለሽ/ በቤት ውስጥ ፅሁፍ የሚያነብልሽ ሰው አለ |  |  |
| 3 | የሞባይል/ የቤት ስልክ አለዎት (የሚሰራ) |  |  |
| 4 | በማህፀን ጫፍ ካንሰር ዙሪያ በዚህ ጤና ጣቢያ መጠይቅ አልተደረገልዎትም |  |  |
| **ማስታወሻ፡** *ከላይ የተጠቀሱት ጥያቄዎች መልስ* ***አራቱም*** *አዎን መሆናቸውን ያረጋገጡ* | | | |

**የጥናቱ መግለጫ እና የተሳተፎ ስምምነት ቅጽ**

በቅድሚያ ጤና ይስጥልኝ እኔ ስሜ **__________________**ሲሆን በዚህ ጤና ጣቢያ የጤና ባለሙያ ሆኜ አገለግላለሁ፡፡ ይህ ጥናት በአዲስ አበባ ዩኒቨርሲቲ ጤና ሳይንስ ኮሌጅ የህብረተሰብ ጤና ትምህርት ቤት የጤና ምርምር ቡድን በማህፀን ጫፍ ካንሰር ቅድመ ምርመራ በተመለከተ እየተሰራ ያለ ነው፡፡ በዚህም ጥናት እንድትሳተፊ በትህትና እንጠይቅሻለን በዚህ ጥናት ላለመሳተፍ መሉ መብት አለሽ፡፡ ለመሳተፍ ከመረጥሽ ደግሞ ቃለመጠየቁን በፈለግሽው ጊዜ የማቋረጥ መብት አለሽ፡፡ ከዚህ ጥናት ላይ በመሳተፍዎ ወይም ባለመሳተፎ ወይም በማቋረጦ ከዚህ ጤና ጣቢያ በሚያገኙት አገልግሎት ላይ ተጽዕኖ አይኖረውም፡፡

በዚህ ጥናት ለመሳተፍ ከተስማሙ ከጤና ጋር የተገናኙ እንዲሁም ስለራስዎ ጥያቄዎችን እንጠይቆታለን፡፡ አጠቃላይ ቃለመጠየቁ 7 ደቂቃ ይወስዳል፡፡

እንዲሁም የጥናቱ ቡድን ከሁለት ወር በኋላ በስልክ ተጨማሪ 3 ጥያቄዎችን ይጠይቋችኋል፡፡

ከዚህ ጥናት የተሰበሰበው መረጃ ሙሉ ሚስጥራዊነቱ የተጠበቀ እንዲሆን እናደርጋልን፡፡ ከጥናት ቡድኑ ውጪ ማንም የተሰበሰበው መረጃ ማግኘት አይችልም እንዲሁም መረጃው ከጥናቱ አላማ ውጪ ለምንም አንጠቀምበትም፡፡ ይህ ጥናት እንዲሳካ የርሶ ፍቃደኝነት እና የነቃ ተሳትፎ አስፈላጊ ነው፡፡

ስለጥናቱ ይበልጥ ማወቅ ከፈለጋችሁ የጥናቱን መሪ ሰላማዊት ሂርጳን እንዲሁም ብርሃን ጣሰውን ማግኘት ይችላሉ፡- +251 911365200/+251911416192

ስለዚህ ጥናት መጠየቅ የሚፈልጉት ነገር አለን?

ለመሳተፍ ፍቃደኛ ኖት ? አዎ አይደለሁም

ለመሳተፍ ፡ ፍቃደኛ፡ ካልሆኑ፡ አመስግነው፡ ይgኙዋቸው ፡፡

**በዚህ ጥናት የሚሳተፉ ሴቶች ሊያ**ሙዋሉዋቸው የሚገቡ ሁኔታዎች

| **ተ.ቁ** | **መስፈርት** | **አዎ** | **አይደለም** |
| --- | --- | --- | --- |
| 1 | ከዚህ ቀደም የማህፀን በር ቅድመ ካንሰር ምርመራ አድርገው ያውቃሉ |  |  |
| 2 | የማህፀን ካንሰር አለብሽ |  |  |
| 3 | ነፍሰጡር ነሽ |  |  |
| 4 | ከወለድሽ 45 ቀን እና ከዛያ በታች ይሆንሻል |  |  |
| **ማስታወሻ፡** *ከላይ የተጠቀሱት ጥያቄዎች መልስ* ***አራቱም*** *አይደለም መሆናቸውን ያረጋገጡ* | | | |

***እነዚህን መስፈርቶች ሁሉም ካልተሟላ መጠይቁን እዚህ አቁሙ

**ክፍል፡ 1 የተጠያቂውንና የጤና ጣቢያው ሙሉ አድራሻ**

1. የተጠያቂዋ ስም _________________________
2. ካርድ ቁጥር_______________________1. የራሷ 2. ሌላ(ይገለፅ)____________
3. ክፍለ ከተማ ________________________
4. የቤት ቁጥር ________________________
5. ሞባይል ቁጥር (የራሷ/ የባለቤትዋ/አማራጭ/የቤት/ካርዱ ላይ የተመዘገበው) 1_________________________2._________________________

3. ________________________(ካርዱ ላይ የተመዘገበውን ቁጥር ይፃፉ)

1. የጤና ጣቢያው ስም________________________
2. የጤና ጣቢያው ኮድ ________________________
3. ቃለመጠይቅ ያደረገው ሰው ስም_________________ፊርማ _________________
4. ቃለመጠየቅ የተደረገበት ቀን **________________________**

**ክፍል 2፡ የተጠያቂው ማህበራዊ ሁኔታ**

**መመሪያ፡ ተጠያቂው የመለሳቸውን መልሶች ከቀረቡት ምርጫዎች ውስጥ** ያክብቡ፡፡

| ተ.ቁ. | ጥያቄ | መልስ | ማስታወሻ |
| --- | --- | --- | --- |
| 101 | የግለሰቧ እድሜ ስንት ነው? | **አመት** |  |
| 102 | የግለሰቧ ሐይማኖት ምንድን ነው? | 1. ኦርቶዶክስ 2. ካቶሊክ 3. ፕሮቴስታንት 4. ሙስሊም 5. ሌላ (ይገለፅ)**_________________** |  |
| 103 | ግለሰቧ ማንበብና መፃፍ ይችላሉ? | 0. አልችልም  1. አዎ | ወደ ጥያቄ 105 ይሂዱ |
| 104 | ግለሰብዋ ያጠናቀቁት ከፍተኛ የትምህርት ደረጃ? | 1. 1ኛ-4ኛ ክፍል ያጠናቀቀ 2. 5ኛ-8ኛ ክፍል ያጠናቀቀ 3. 9ኛ-12ኛ ክፍል ያጠናቀቀ 4. ዲፕሎማ 5. 1ኛ ዲግሪ   6. ማስተርስ እና ከዛ በላይ |  |
| 105 | የግለሰቧ ዋነኛ ስራ ምንድን ነው? | 1. የቤት እመቤት 2. የመንግስት ሠራተኛ 3. የግል ድርጅት ሠራተኛ 4. ነጋዴ 5. የቀን ሠራተኛ 6. ገበሬ 7. ተማሪ 8. ሌላ(ይግለጹ)_________________ |  |
| 106 | የግለሰቧ የጋብቻ ሁኔታ ምንድን ነው? | 1. ያላገባ 2. ያገባ 3. የፈታ 4. የተለያየ 5. የትዳር አጋር በሞት የተለየ | መልሱ **1፣3፣4፣5** ከሆነ ወደ ጥያቄ**109**ይሂዱ |
| 107 | የግለሰቧ ባለቤት ማንበብና መጻፍ ይችላሉ | 0. አይችልም  1. አዎ | ወደ ጥያቄ 109 |
| 108 | የግለሰቧ ባለቤት ያጠናቀቁት ከፍተኛ የትምህርት ደረጃ? | 1. 1ኛ-4ኛ ክፍል ያጠናቀቀ 2. 5ኛ-8ኛ ክፍል ያጠናቀቀ 3. 9ኛ-12ኛ ክፍል ያጠናቀቀ 4. ዲፕሎማ 5. ዲግሪ 6. ማስተርስ እና ከዛ በላይ |  |
| 109 | በአባላዘር በሽታ ታመው ያውቃሉ? | 1. አያውቅም 2. ያውቃል |  |
| 110 | የኤች አይ ቪ ምርመራ ተደርጎሎት ያውቃል? | 1. አላውቅም 2. አዎ | ጥያቄውን እዚህ ያቁሙ |
| 111 | የኤች አይቪ ምርመራ ውጤትዎ ምን ነበር? | 1. ኔጌቲቭ 2. ፖሰቲቭ 3. መናገር አልፈልግም |  |

**የጥናቱ ተሳታፊ የመጀመሪያ**ውን መጠይቅ ከተደረገላቸው ከሁለት ወር በኃላ በስልክ የሚጠየቁት ጥያቄዎች

| 1. | ባለፈው 3 ወር ለማሕፀን በር ቅድመ ካንሰር ምርመራ አድርገሻል | 1. አይደለም 2. አዎ | ጥያቄ 3 ይሂዱ |
| --- | --- | --- | --- |
| 2. | መልሱ አላደ(1) ኩም ከሆነ ምክንያቱን ይግለè፡፡ | 1.ታምሜ አላውቅም ለምን ያስፈልገኛል  2. ምርመራው የሚካሄድበትን መንገድ አልወደውም  3.ግልጋሎቱ የት እንደሚሰጥ አላውቅም  4. ስለዚህ ነገር የማውቀው ነገር የለም  5. ሌላ ይገለፅ)**______________** | |
| 3 | የቅድመ ካንሰር ምርመራ እንዲያደርጉ የረዳዎትን መረጃ ከየት ነው ያገኙት ? | 1. 1.ራዲዮ ወይም ቴሌቪዥን 2. ዘመድ ወይም ጓደኛ 3. የጤና ባለሙያ 4. የጤና ባለሙያ እና በፅሁፍ ያገኘሁትን ትምህርት 5. . ሌላ ይገለፅ)**______________** | |
| 4 | የቅድመ ካንሰር ምርመራ ውጤት ? | 1.ኔጌቲቭ  2. ፖሰቲቭ ( በዚህ ጤና ጣቢያ ታክመዋል)  3. ፖሰቲቭ ( ወደ ሌላ፡ ምርመራ ወይም ህክምና ተልከዋል)  4. አላውቀውም | |

**Annex 2**

**Educational material (Adopted from Ministry of Health brochure on important health messages)**

**የማህጸን፡ በር ፡ ካንሰር ፡ ምንድነው?**

ይህ፡ የካንሰር፡ አይነት፡ በኢትዮጵያ ፡ ለህመምና ፡ ለሞት፡ ከሚዳርጉ፡ የሴቶች፡የካንሰር፡ አይነቶች፡ ውስጥ፡ አንዱና፡ ዋናው፡ ነው፡፡ የማህጸን፡ በር ፡ ካንሰር፡ ሂውማን፡ ፓፒሎማ፡ ቫይረስ፡ በተሰኘ፡ ረቂቅ፡ተህዋስ፡አማክኝነት፡የሚከሰት፡ ነው፡፡ ተህዋሱ፡ የማህጸን፡ በር ፡ ህዋሳትን፡ በመውረር፡ ጤናማ፡ ያልሆነ፡እድገትና፡ ብዜትን፡እያስከተለ፡ወደ፡ ሌሎች፡አካላት፡ክፍሎች፡በመሰራጨት፡ ለህመምና፡ ለሞት፡ የሚዳርግ፡ ነው፡፡

የማህጸን፡ በር፡ ካንሰር፡ ስር፡ እስኪሰድ፡ ድረስ፡ ምንም፡ አይነት፡ ስሜትና፡ ምልክት፡ ሳይኖረው፡ 15-20 አመት፡ሊቆይ፡ይችላል፡፡ ከተባባሰ፡ በኃላ፡ ግን፡ ደም፡ በብልት፡መፍሰስ፤ ያልተለመደ፡የማህጸን፡ ፈሳሽ፤ የማህጸን፡ አካባቢ፡ ህመም፡ እንዲሁም፡ በግብረ፡ ስጋ፡ ግንኙነት፡ ወቅት፡ ህመም፡ ሊሰማ፡ ይችላል፡፡

የማህፀን

በር

**የማህጸን፡ በር ፡ ካንሰር ፡ተጋላጭ፡የሆኑ፡ እነማን፡ ናቸው፡**

ማንኛውም፡ የግብረ፡ ስጋ፡ ግንኙነት፡ ፈጽማ፡ የምታውቅ፡ ሴት፡ ሁሉ፡ የማህጸን፡ በር ፡ ካንሰር ፡ተጋላጭ፡ናት፡፡ ይሁን፡ እንጂ፡ ከ 20 አመት፡ በታች፡ የግብረ፡ ስጋ፡ ግንኙነት፡ መጀመር፣ከተለያዩ፡ ወንዶች፡ ጋር፡ የግብረ፤ ስጋ፡ ግንኙነት፡ መፈጸም፣ ከብዙ፡ ሴቶች፡ ጋር፡ የግብረ፤ ስጋ፡ ግንኙነት፡ ከሚፈጽሙ፡ ወንዶች፡ ጋር፡ የግብረ፤ ስጋ፡ ግንኙነት፡ያላቸው፡ ሴቶች፣በተለያየ፡ ምክንያቶች፡ የሰውነት፡በሽታ፡ የመቋቋም፡ ሀይል፡ መቀነስ፡ ያጋጠማቸው፡ ሴቶች፡ ለምሳሌ፡ ኤች.አይቪ ቫይረስ፡ በደማቸው፡ ውስጥ፡ ያለ ፣በአባላዘር፡ በሽታዎች፡ የተያዙ፡ እና፡ ሲጋራ፡ የሚያጤሱ፡ ሰዎች፡ በይበልጥ፡ ተጋላጭ፡ ናቸው፡፡


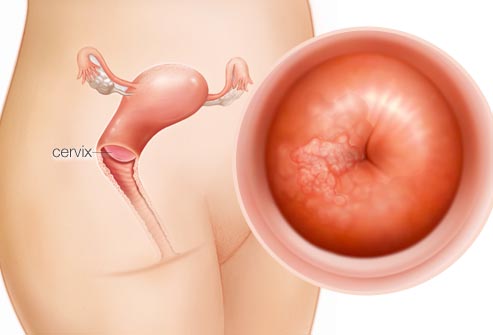


የማህፀን በር

**የቅድመ፡ ካንሰር፡ ምርመራና፡ ህክምናውስ**

የማህጸን፡ በር፡ ካንሰር፡ መስፋፋት፡ በጣም፡ አዝጋሚ፡ በመሆኑ፡ ቅድመ፤ ካንሰር፡ ምርመራ፡ በማድረግ፤ በሽታው፡ ተባብሶ፡ለህይወት፡አስጊ፡ ከመሆኑ፡ በፊት፡ ታክሞ፡ መዳን፡ ይቻላል፡፡ የማህጸን፡ በር፡ ካንሰር፡ መኖር፡ ያለመኖሩን፡ ለመለየት፡ የተለያዩ፡ ምርመራዎች፡ ይደረጋሉ፡፡

ለምሳሌ፡ የቪአይኤ፡ ቅድመ፡ምርመራ፡ በዚህ፡ ጤና፡ ጣቢያ፡ ይገኛል፡፡

በመሆኑም፡ የምርመራው፡ ውጤት፡የቅድመ፡ካንሰር፡ ምልክት፡ ካሳየ፡ በህክምና፡ ለማደን፡ ይቻላል፡፡ ይህ፡ የህክምና፡ አይነት፡ ክራዮቴራፒ በመባል፡ የሚታወቅ፡ ሲሆን፡ የማህጸን፡ በር፡ ካንሰር፡ ከመከሰቱ፡ በፊት፡ 90 በመቶ፡ በላይ፡ ይከላከላል፡፡ ስለዚህ፡ ከ30-49 አመት፤ የሆናቸው፡ ሴቶች፡ በየአምስት፡ አመቱ፡ አንድ፡ ግዜ፡ ቅድመ፡ ምርመራ፡ ማድረግ፡ ይኖርባታል፡፡


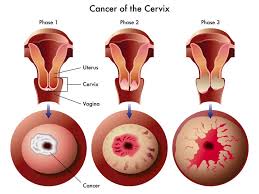


የማህፀን በር ካንሰር

ደረጃ 1

ደረጃ 2

ደረጃ 3
