# Supplementary material for: The role of health education on cervical cancer screening uptake at selected health centers in Addis Ababa
Source: PLoS One. 2020 Oct 7;15(10):e0239580. doi: 10.1371/journal.pone.0239580 (PMC7540882; doi:10.1371/journal.pone.0239580)
Supplement: S2 File — (DOCX) [file pone.0239580.s003.docx]

**Information Sheet for the Intervention Group**

My name is …………………………………. I am here on behalf women research group of Addis Ababa University School of public health. We are conducting a research entitled “Health Information dissemination and Print Media to enhance cervical cancer screening”.

You are selected randomly by chance. We kindly invite you to participate in this study. Your participation is purely based on your willingness. You have the right to choose not to take part in this study. If you choose to take part, you have the right to stop the interview at any time. If you are willing to participate or refuse or decide to withdraw later, you will not be subjected to any ill-treatment.

If you agree to participate in the study, you will be asked to answer some questions about health-related questions and yourself. The interview will be done twice; the first interview is today and the next will be after two months through telephone. The current interview will take 20 minutes. After the first interview, health education and print media will be given to you about cervical cancer screening.

This study will have a contribution to increase the uptake of cervical cancer screening. It can also provide base line data for policy makers and other researchers for further studies. The information that you provide will be kept confidential by using only code numbers and locking the data. No one will have access to the non-coded data except the principal investigators and the data will not be used for purposes other than the study. Your willingness and active participation is very important for the success of this study.

If you want to know more about the study, you can contact the investigators Selamawit Hirpa and Berhane Tassewe +251 911265200/+251911416192

Do you have any question?

**Consent form**

Are you willing to participate yes

No

If you are not willing to participate thank you .

| No. | Requirements to proceed | Yes | No |
| --- | --- | --- | --- |
| 1 | Age 30- 49 |  |  |
| 2 | Can you read or do you have someone who can read for you |  |  |
| 3 | Do you have mobile phone or any phone that we can reach you |  |  |
| 4 | Have you been screened for cervical cancer previously |  |  |
| 5 | Have you been interviewed on cervical cancer screening in these facility |  |  |
|  |  |  |  |

N.B – If the respondent says ‘No’ to one of the above questions; will not be included in this study

Part I: Participants health facility full address

1. Card number _____________________1. her Owen 2. Other(specify) ____________
2. Sub city ___________________________
3. House number ______________
4. Mobile number (Private /her husbands or any other number written in the card
5. _____________ 2. __________________ 3. ______________(from the card)
6. Name of the health facility ______________
7. Health facility code ______________
8. Interviewer name ___________________________signature _____________

Interview date ______________________________

**Instruction**: circle all the possible answers of the respondent from the choice provided

Part 2- **Socio-demographic characteristics of participants**

| **Q.No.** | **Question** | **Choices** | **Remarks** |
| --- | --- | --- | --- |
| 101 | How old are you? (age in years) | **አመት years** |  |
| 102 | What is your religion? | 1. Orthodox 2. Catholic 3. Protestant 4. Muslim 5. Other specify__ |  |
| 103 | Can the participant read and write | 1. No 2. Yes |  |
| 104 | Highest level of education obtained | 1. Grade (1-4th) 2. Grade (5-8^th^) 3. Grade (9-12th) 4. Diploma 5. First Degree 6. Masters and above |  |
| 105 | Participant’s occupation | 1. Housewife 2. Government employee 3. Private employee 4. Merchant 5. Daily labourer 6. Farmer 7. Student   88. Other **(specify)_____________** |  |
| 106 | What is your current marital status? | 1. Single 2. Married 3. Divorced 4. Separated 5. Widowed | **If the answer is 1, 3, 4 or 5 skip to question number 109**. |
| 107 | Partner’s educational level | 1. Can’t read and write 2. Can read and write | If answer is 1 go to question 109 |
| 108 | Partner’s educational status? | 1. Grade (1-4th) 2. Grade (5-8^th^ ) 3. Grade (9-12th) 4. Diploma 5. Degree 6. Masters and above |  |
| 109 | Did you have history of STI? | 1. No 2. Yes |  |
| 110 | Have you ever had HIV/AIDS test | 1. No 2. Yes | If NO stop here |
| 110 | If you have been tested, what was the result? | 1. Negative 2. Positive 3. I don’t want to tell |  |

**Participants second round interview after two months with phone call**

| **Question number** | **Questionnaire** | **Response** | **Skip** |
| --- | --- | --- | --- |
| **1** | Have you been screened for cervical cancer in past two months? | 1. No 2. Yes | **If ‘Yes’ go to question number 3** |
| **2** | If no to the above question what is the reason? | 1. I have never been sick why do I need it 2. I don’t know how the test is done 3. I don’t know where the service is given 4. I don’t know anything about this 5. Specify if other ________ |  |
| **3** | Where did you get information to do the cervical cancer screening | 1. From radio or television 2. Family and friends 3. Health professionals 4. Health professionals and the written information I got 5. Specify if other _______ |  |
