# Supplementary material for: The role of health education on cervical cancer screening uptake at selected health centers in Addis Ababa
Source: PLoS One. 2020 Oct 7;15(10):e0239580. doi: 10.1371/journal.pone.0239580 (PMC7540882; doi:10.1371/journal.pone.0239580)
Supplement: Attachment — Submitted filename: Response (2).odt [file pone.0239580.s004.odt]

Research Title- The Role of Health Education on Cervical Cancer Screening Uptake at Selected Health Centres in Addis Ababa

Responses 

Dear Editors thank you for your valuable comments. I have responded and considered your comments.  
Comment- In the Methods, please clarify that participants provided oral informed consent. Please also state in the Methods:
We have now included in the Methods section

Comment - Why written consent could not be obtained
The study participants were in different educational status including women who could not read and write.  Mostly the illiterate individuals in Ethiopia are not comfortable to put any signature on a document which they did not read by themselves.  With these reasons we preferred the oral consent. The oral consent was taken under the full knowledge of IRB and we followed standard ethical guideline 

- How oral consent was documented
All women in the age range of 30-49 who went to the study areas specific departments were informed by trained health professionals about the study. The health professionals took a training including the ethics issues.  The information included the purpose of the study, all information needed for decision to participate or not, how they are selected for the study, voluntary participation, the right to refuse to answer any question, issue of confidentiality.  After that the data collectors asked orally whether they were willing to participate or not. The data collector ticked their responses in the box provided on the consent form.  


(2) Thank you for the ethics statement:

'Ethical clearance was obtained from the Addis Ababa University College of Health Sciences Institutional Review Board'.

Comment - At this time, we ask that you please state that the IRB specifically approved the study
Response- At Addis Ababa University College of Health Sciences, there is one institutional review board and it does not have specific names.
