# Supplementary material for: The role of health education on cervical cancer screening uptake at selected health centers in Addis Ababa
Source: PLoS One. 2020 Oct 7;15(10):e0239580. doi: 10.1371/journal.pone.0239580 (PMC7540882; doi:10.1371/journal.pone.0239580)
Supplement: Attachment — Submitted filename: Response for reviewers (version 3).odt [file pone.0239580.s005.odt]

Response for reviewers

Dear reviewers, 
Thank you so much for the constructive comments. We have addressed your comments one by one below 
Reviewer #3
Comment-: This study examined the effect of health education on cervical cancer screening uptake in 2,139 women at selected health facilities in Addis Ababa, Ethiopia, where awareness of the disease and availability of screening and treatment are limited. A RCT was conducted at eight public health care centers that provide cervical cancer screening services using visual inspection with acetic acid; each center was assigned to either intervention (one-on-one health education, and receipt of a brochure explaining cervical cancer and benefits of screening) or control (regular care). After two months, women were contacted to ask whether they had been screened for cervical cancer.

This paper deals with a very important topic, and an easily and inexpensively implemented intervention, but given the results, it is NOT effective. The authors found a significant effect in the intervention group; however, they need to address more fully the obvious problem that even in the intervention group, there was only 14% uptake of services. The intervention may work better than nothing, but it isn’t working very well if >85% of the women who received the intervention did not seek screening. Although the authors surmise what the reason for the low rate of uptake might be, they do not take the opportunity to put forward ideas about what might have been done differently or what might have been wrong with the intervention.

Was any attempt made to schedule an appointment for the women at the time they were enrolled, or at any time after the intervention? Education helped but was in no way enough to motivate these women; it either missed the mark in the way the information was conveyed, or did not include enough information to be taken seriously. Scheduling an appointment on the spot would be far more effective than leaving it up to these women themselves, who are older and therefore probably assume they have managed to go without screening this long without any bad effects, so “why bother?”

At two months, the majority of the women in the intervention group (almost 80%) said that the primary reason for not seeking screening was because they “were busy;” in person, this sort of excuse could be directly addressed. The majority of the women are housewives, with presumably older children (given their age range). If the intervention included an attempt to schedule an appointment, perhaps this “too busy” issue could be directly addressed with these women and solutions found (were they really “too busy,” or did they just need further nudging, reassurance and a stronger emphasis on the importance of screening?). If this study is to be valuable to future researchers, the authors should put more emphasis on what went wrong, why, and how it might be fixed.

The study needs to be edited by a native English speaker; there are many incomplete or incoherent sentences and grammatical errors.

Response
- There was no attempt made to schedule an appointment for the women at the time they were enrolled or at the time of intervention. It is true that arranging a schedule for a woman increases the turnout for screening. The study participants were women who visited government health centres for family planning, immunization service, or children clinic during the study period. The VIA screening room was a different room and managed by different health professionals. The outpatient departments were not computerized and networked. If our intervention includes arranging a schedule, the health professional should go to the VIA room with each patient to facilitate that. This might be doable for the research purpose but not feasible in a real-life scenario with a country where health professionals have high client/patient load.  
- Now we have elaborated more why most the women in the intervention group didn’t get screened (Please see discussion section – Paragraph 4)

Reviewer 4  
Comment - Abstract:
[Methods]
“The eight health centers were stratified into two equal numbers of groups as intervention and control.” A more clear way to state this would be: Each of the eight health centers was randomly assigned to serve as either an intervention or a control centre.
Response: We have accommodated the comment  
Comment :Introduction
Delete the reference to global cases of cervical cancer; it isn’t really relevant. Should just read, “In 2018 alone, 6,294 new cases of cervical cancer occurred in Ethiopia…”
Response: Comment accommodated 

Comment Methods
A table for the sample size calculation really isn’t necessary. This information can be included in the text.
[Data entry and analysis] First sentence should read “Data entry and data analysis were done using…. respectively.”
Response- Comment accommodated 

Comment 
Table 1.
The authors should consider testing the differences between the two cohorts, given that the groups each come from four different centres.
Response - We have done a Chi- square test result of cervical cancer screening practice for intervention and control health facilities 
Comment 
Table 2.
Could the authors address whether they had difficulty contacting the women after two months? It appears from Table 2 that follow-up was very good but not complete. What efforts were made to contact the women who could not be reached? The authors should summarize this information in the results (including reporting the percentage of incomplete assessment in each group). It would be interesting to compare statistically the reasons that women gave for not being screened between the two groups (simple chi-square tests could be used).
Response – In the result section first paragraph, now we have tried to address the comments.  For reasons for not being screened , we have done a Chi- square test  ( Refer -table 3)

Comment 
Table 3.
The title of the table should not be “Multivariable Analysis…” if it also contains the bivariate analysis (I am assuming this is what the “Crude OR” refers to). The unadjusted analyses should show results of all models, even those for covariates that did not turn out to be significantly associated with the outcome. The columns for variables not included in the multivariable analysis would just be left blank in the “Adjusted OR” columns. Group status should be listed first. P-values should be reported for both the unadjusted and adjusted models. There is a “1” missing (as reference) for Marital Status for Crude OR.
Response 
Comment accommodated (Refer Table 4)

Comment 
Discussion
7% of the intervention group reported that they were not screened because they didn’t know about cervical cancer screening and availability of the services. This is surprising in the group that received the intervention and indicates some failing of the intervention for them. Did the authors pursue this, to determine why these women claimed to lack awareness?
Response – This is a valid point.  But this was expected since almost 14 % of the study participants in the intervention group was illiterate. They might not fully benefit from  a one to one brief  health education and educational brochure.  We didn’t further investigate why these women claimed to lack awareness. 


Comment 
ABSTRACT
Several phrases in the abstract or in the underlying main article are unclear and are noted below

Methods
one-to-one brief health education: Need further description. The One to one brief health education in the interventional centre is obvious. How does health education in the interventional centre differ from the non-interventional (control) centre? A clearer description is needed describing the differences of the client receives at the interventional vs non-interventional centres.

Response- we have addressed the comment (Refer- Methods part, control section)

Comment pectoral description : “Pectoral” refers to the chest muscles; I believe the authors mean “pictures.”
Response – We have accommodated the comment

Comment- After two months: Did clients sometimes have the treatment at the time of the initial visit or did they actually have to go home and then return for a special cancer screening

Response – Our study did not record this. But both was possible; most women who came for immunization service or with sick children might prefer to come other time for the cervical cancer screening 

Comment 
called through their phones: Awkward. Called by phone somehow sounds better
Response- Thank you, comment accommodated 
Comment 
Results
Comment- 215 (10%) : Medical care in Addis Ababa. The intervention essentially doubled the number of people screened for cervical cancer, yet the overall rates remained low (10% of all women in the study). The goal, ideally, is for a 100% rate of screening. Am I correct only about 3% (64) of the non-interventional cluster came for screening? The discussion section of the paper might wish to offer thoughts as to why the rates for both groups remained so low.

Response- Now this comment is addressed in the discussion part; paragraph 4. 

Comment - first degree or above: To what does first degree refer: education?

Response-Comment accommodated 
Comment- STI: Spell out
Response – Comment accommodated 

Comment 
Introduction
3rd Paragraph: The expansion …
study done among 1186 women in Mekelle : Interesting that the rate of screening at Mekelle was roughly 6 times greater there than at your institution (19.6% vs 3% of your noninterventional cluster). What is the difference in your two populations
Response – In the Mekelle study, they have done community-based study whether they have a history of cervical cancer screening. In this study we have excluded those who have a history of cervical cancer screening. We have followed women who have no history of cervical cancer screening in the intervention and control group whether they have screened for cervical cancer or not. 
Comment 
5th para: In our study …
theoretical : I do not believe you mean “theoretical,” more likely the word should be “practical”
Response - Health Belief model is a theoretical model 
Comment 
Methods
Study area and period
The study was conducted in Addis Ababa, the capital city of Ethiopia between August 2017 and January 2018.: the sentence is constructed awkwardly. The authors mean the study was conducted between August 2017 and January 2018, but as written states that Addis Abba was the capital between those two dates.
Response- Comment accommodated 

Comment: tire: Tier, not tire
Response: Comment accommodated 

Comment- Unlike other regions in the country,: Why are the Addis Ababa health centers unlike those of other parts of the country? Do the others lack general practitioners, nurses, or midwives? Also, for those of us unfamiliar with the health system in Ethiopia, what are the key characteristics of the second level and tertiary level care providers?

Response – In the other regions in the country, there is no general practitioners in the health centres.  The difference between second level and tertiary level hospitals is, the tertiary hospital is specialized referral and teaching hospital. 

Study design and population
Comment- Two-pronged a cluster: Do you mean “a two-pronged” rather than “two-pronged a”?

Response- Comment accommodated 

Comment - 30-49 years. Which is: I believe you mean “30-49 years, which is” rather than a new sentence “30-49 years. Which is”

Response – Comment accommodated 

Comment 
under 5 clinics for their babies : I think you mean “babies and children under five years of age”

Response – It is corrected to clinics for children under five years of age   

Comment - Women who gave birth in the past 45 days and pregnant: awkward wording. The sentence reads as if someone had recently given birth but is still pregnant.
Response – Now, we have corrected to “Pregnant women and women who gave birth in the past 45 days during the study period.

Comment  
Intervention group:
one-to-one brief health talk : Given one to one talk. How was the brochure the interventional group received different from that the control clients received? Within the interventional group, what was more effective, the personal talk, or whatever reading material the client received?

Response: In the control group, they didn’t receive brochure. This study measured both the effect of brief health talk and educational brochure

Comment  

Each woman who participated in the intervention group: Would it be correct to assume that women in the intervention group did NOT have the screening performed at the same time as they came for the initial interview? Did many of the women at the initial interview schedule their return visit for VIA or did they return home, and once there, schedule a visit? This seems to me to be important in determining what type of a future educational campaign might be waged. If the former, more women need to be encouraged to come to the centres and have one to one interview. If the latter, then the campaign may consist more of brochures sent out throughout the community
Response: It would be correct to assume that most women in the intervention group did not have the screening performed at the same day as they came for initial interview. The reason for this is, most of the women came to the health centres for immunization and clinics for under 5 years of age. It is unlikely that women who came to health centres with sick child or a baby under one years of age would be screened at the same day. 
So, it means women return to their homes came again in any day (within 2 months’ time from the study period) to the health centres for the reason to be screened.  

Comment 
Control group:
except the regular health education: It is unclear of what “regular health education” consists.
What exactly is the difference between “regular” in the controls and the brochures in the interventional group. This would seem to be an important facet to clarify in a study of this type. Some more detail would be helpful.

Response: In the control group, the regular health education program is given by the health professionals in the morning before  clinics start to work. Patients or clients who stay in the waiting area to get the health service would be the target for the health education.  In the package of health education, cervical cancer is given at least twice a week in the health centres under study. (Refer, Method section, In control group)

Ethical consideration:
Comment 
Each respondent was informed about the purpose, scope, and expected the outcome of the research and verbal informed consent as it was approved : Unclear: Were control told they would not be given 1-1 counselling?
Response – We did not tell them that they would not be given one to one counselling. In general terms, we have said that the study will inform policy makers and further researches. Also, we have said, if they are willing to participate there will be second round interview which will be conducted through telephone interview. 

Comment 
The data collectors put a mark on the checklist given on the consent form, regarding the study participants' responses to participate. : Are the authors’ saying checkmarks were put on all clients forms, so that at the end of the study the authors could determine the total number asked, and thus also what percent of those asked to join the study, regardless whether intervention or control, actually refused? I presume that is from where the number, 2139, derives.

Response – We had a separate form to record the percentage of women who were not willing to participate or excluded from the study. The number 2139 derives from the lost follow up ( The second round of interview was conducted through telephone and at that time we were unable to contact some of them)-  At initial time 2, 400 participants were recruited in the study and after two months of time( For the follow up interview) when we tried to contact them , we could reach only 2,140. (We have put this on the first paragraph of the result section) 
N.B The number 2139 is now corrected to 2140 

Comment 
The study is also hazy about what “participates” means, which affects the study numbers, and hence percentages reported.
Response- Now, we have corrected to recruited study participant 

Comment- 

When a woman came to Bole-17 for example, was she asked if she would participate, and if so, only then did she receive a one-on-one session?
Response- Yes. Also, we also checked that she fulfilled the screening criteria 

Comment 
Also, was VIS performed on the same day and possibly even at the same time as the interview, or did clients have to return on a subsequent day, which certainly would reduce compliance and therefor the number of study participants completing all desired aspect?
Response- In the intervention group, we have given the health education and brochure for the recruited women in this study. We have conducted the second-round interview after two months whether they were screened for the cervical cancer or not. We did not arrange any schedule for screening. 

Result
Comment 
A total of 2,139 women who had never screened: Add the missing word “been” after “never”
Response – Comment accommodated 

Comment 
During the study period, until this point, the two populations seem relatively similar. The rest of the paragraph left me stunned, and unprepared to understand how the population as a whole differed from both the interventional and control clients. (Actually, the answer came only late in the discussion – ALL pregnant women are routinely tested for HIV. Thus, nearly 100% of the clients have been tested for HIV. Is this normal for Africa, for Ethiopia, specifically for Addis Ababa, or specifically for the population of patients with which your group deals? Somewhere earlier, there needs to be a description, however brief, of your population.

Response – HIV testing is a routine program for Antenatal care (ANC programme) in Ethiopia.  In this study, the participants are in the age group of 30-49 years. Almost all have visited ANC service at least once in their life time. 
Comment 
The remainder of the paragraph should present the data to help the reader. Possibly the authors might wish to present the HIV data, then the STI data and finally the cervical data.
Response: Comment accommodated 
Comment 
In the discussion, somewhere, you may wish a few comments about how your group is forward-looking and trying to advance medical care in Ethiopia in ways that are economically practical.
Response – In the recommendation part, we have said that integrating a one-to-one health education and administering educational brochures which are written in simple languages about cervical cancer and benefits of early screening with the existing maternal health programs are worthwhile.
Comment 
Regarding the health status of participants, My comments refer to this paragraph and also table 1. The single most striking fact of table 1 is that over 90% of all your clients, regardless of whether intervention or control, were tested for HIV, now or in the past. From the discussion, I presume this is routine.

With this high rate of HIV testing, why is there not greater concern about STI's and cervical cancer? It seems cervical cancer is relatively ignored. Is this because the women generally know nothing about cervical cancer and do not understand it? Is this part of the overall education programs your medical centers/leaders are trying to bring to the Ethiopian citizenry? The discussion might expand on this subject.
Response- HIV testing  and STI screening is a routine programme for the antenatal care (ANC).  We have accepted the comment and expanded the discussion part in paragraph 2 

STI. Where 1,005(95%): Spell out STI as it is the first time of use in the text.
I believe the two phrases belong to one sentence.
Response- Comment accommodated 

Table 1. Socio demographic and Health Related Information of Study Participants at Addis Ababa, 2017
Diploma
First Degree and above: What is the difference between diploma and first-degree; Will most readers understand?
Response – Now, we have changed “Diploma” to technical and vocational training 

Comment 
Religion
Orthodox : Are not the orthodox part of the Christian community? Would it be clearer, given a worldwide readership likely unfamiliar with Ethiopian religion to refer to the Orthodox as Ethiopian Christian Orthodox? This would avoid confusion with Jewish Orthodox.

Response - Comment accommodated 
Comment 
All study participants … telephone about the source of information after two months of time from the recruitment period: This sentence is problematic. The study has two arms where patients received specific one-on-one counseling and brochures, or little other than the general information provided by the clinic. But now a third arm is suggested, where the patients receive new sources of information during the subsequent telephone calls. Does this mean some clients in the interventional or control groups learn from a third source and were acting on that new information? Do we now have a murky third arm? Please explain.
Response – There was no third arm of group or no counselling was given through telephone for any of the groups.  After two months of time from the recruitment period, all study participants were interviewed through telephone.  

Discussion
Comment 
This study was conducted in an urban area where most people can read and write or have someone around to read for them : Useful in methods to help describe the population clientele.
Response: Comment accommodated 
Comment 
Paragraph:
In this study, women who were screened for cervical cancer reported that the health education they have received from one-to-one health education and the educational brochure had helped them to decide in screening for cervical cancer. : Was there anything the healthcare professionals learned when giving advice to getting the clients attention and having the client follow through with testing? If so, please add in the results section and comment in the discussion section.
Response -  Our study only focused on women who had visited the health centres

Comment 
Paragraph: This study also found a significant association between history of STI
. This could be related to the fact that HIV screening is a routine procedure that is provided for all pregnant women at all health facilities hence: I was unaware of this fact, which explains my comment related to table 1. Please comment about routine HIV screening in pregnant women much earlier.
Response- Comment accommodated 
Comment 
References
: Please refer the Journal’s instructions; Most references are incomplete
#2 : Who is the author, what is the journal or book etc.
#3 : Is this a web reference. Need URL & Date access
#4 : Web site note proper – too many periods.
#5 : Citation incomplete
Etc.

Response – Comment accommodated
